# Identifying an efficient, thermally robust inorganic phosphor host via machine learning

**DOI:** 10.1038/s41467-018-06625-z

**Published:** 2018-10-22

**Authors:** Ya Zhuo, Aria Mansouri Tehrani, Anton O. Oliynyk, Anna C. Duke, Jakoah Brgoch

**Affiliations:** Department of Chemistry, University of Houston, Houston, TX 77204 USA

## Abstract

Rare-earth substituted inorganic phosphors are critical for solid state lighting. New phosphors are traditionally identified through chemical intuition or trial and error synthesis, inhibiting the discovery of potential high-performance materials. Here, we merge a support vector machine regression model to predict a phosphor host crystal structure’s Debye temperature, which is a proxy for photoluminescent quantum yield, with high-throughput density functional theory calculations to evaluate the band gap. This platform allows the identification of phosphors that may have otherwise been overlooked. Among the compounds with the highest Debye temperature and largest band gap, NaBaB_9_O_15_ shows outstanding potential. Following its synthesis and structural characterization, the structural rigidity is confirmed to stem from a unique corner sharing [B_3_O_7_]^5–^ polyanionic backbone. Substituting this material with Eu^2+^ yields UV excitation bands and a narrow violet emission at 416 nm with a full-width at half-maximum of 34.5 nm. More importantly, NaBaB_9_O_15_:Eu^2+^ possesses a quantum yield of 95% and excellent thermal stability.

## Abstract (web summary)

Identifying phosphors with good thermal stability and quantum efficiency is a prerequisite to improve the performance of white LED light sources. Here, a combined machine learning and density functional theory method is introduced to identify next generation inorganic phosphors.

## Introduction

Inorganic phosphors are among the most significant components in a light-emitting diode (LED)-based white light and greatly influence the device’s overall efficacy^1–3^. These materials are composed of a host crystal structure, often an oxide, halide, or nitride, that are substituted with a rare-earth luminescent center, typically Eu^2+^ or Ce^3+^^4–7^. The rare-earth ion absorbs at least part of the LED emission and then re-emits a photon at a longer wavelength. The combination of these emissive lights cover the entire visible portion of the electromagnetic spectrum and thus appears as white light^3,8^. The efficiency, color quality, and compact size make these devices not only useful for general illumination, but they are also essential for modern display applications^9^. Unfortunately, only a handful of the phosphors currently reported in the literature are able to perform the down-conversion process efficiently, as measured by the photoluminescent quantum yield (*Φ*)^10^. Even then, some of the phosphors with a high luminescence quantum efficiency also suffer from thermal quenching, where the optical properties, for example, emission wavelength and intensity, change at elevated temperature limiting their range of potential applications^11–15^. Therefore, the continued improvement of LED-based lighting requires the discovery of new efficient and thermally robust rare-earth substituted inorganic phosphors.

In the search for new phosphors, research has shown one viable method for identifying materials with a high *Φ* is to find structurally rigid host compounds with high atomic connectivity^16^. These characteristics enhance *Φ* by inhibiting soft phonon modes that lead to non-radiative relaxation, thereby increasing the propensity for photon emission. However, the complex nature of chemical bonding in inorganic solids makes clearly identifying connectivity and structural rigidity challenging, and the comparison of rigidity between multiple solids problematic. Alternatively, it is possible to use a proxy, which is a quantifiable metric that scales with structural rigidity, to compare different materials. Prior work has already shown that a material’s Debye temperature (*Θ*_D_) is the most reliable proxy for structural rigidity, and consequently useful for screening *Φ*^17–19^. Materials with a higher *Θ*_D_ tend to have higher-energy phonon modes that decrease the probability of non-radiative relaxation increasing *Φ*, whereas materials with a low *Θ*_D_ tend to contain softer phonon modes that can promote non-radiative relaxation. More importantly, *Θ*_D_ can be estimated using density functional theory (DFT), allowing for the potential of high-throughput screening^20^. Nevertheless, this approach has numerous drawbacks; most notably, calculating *Θ*_D_ using DFT is computationally arduous. DFT cannot easily account for atomic disorder like site sharing which is common in complex inorganic solids, and it is currently restricted to smaller unit cells, typically a few hundred atoms at most. These limitations have permitted the elastic moduli, which are necessary to estimate *Θ*_D_, to be calculated for only a few thousand compounds, or <10% of the reported inorganic solids.

Advances in machine learning provides an avenue to significantly expand the physical and mechanical properties of inorganic solids and can push materials development beyond the current capability of DFT alone^21,22^. For example, it is possible to use a kernel ridge regression model to predict a myriad of electronic properties including dielectric constants, electron affinities, formation energies, and even band gaps^23,24^. A more intricate method employing a gradient boosting decision tree and universal fragment descriptors is able to predict the electronic as well as mechanical properties, such as bulk and shear modulus, heat capacity, and Debye temperature^25^. Machine learning has also allowed researchers to predict glass formation^26^, study magnetoelectric heterostructures^27^, and optimize microstructure^28,29^. The great advantage of using statistical machine-learning methods is that predictions can be quickly made for any given combination of elements, any stoichiometry, or any size unit cell. We show here that it is also possible to use machine learning to predict *Θ*_D_ for a majority of compounds in the Pearson’s crystal database (PCD)^30^ in seconds regardless of unit cell size, atomic mixing, or electron correlation resulting in ~120,000 Debye temperatures that can be used for screening inorganic phosphors.

Knowledge of a crystal structure’s *Θ*_D_ alone is still not sufficient to produce a high photoluminescence quantum efficiency inorganic phosphor. A wide band gap (*E*_g_) in the host crystal structure is also crucial^20^. The size of the band gap sets the relative position of the rare-earth 5*d* orbitals with respect to the host crystal’s conduction band, which can influence the optical response. If the band gap of the host is small, the excited-state 5*d* orbitals may be close enough to the conduction band of the host crystal structure to allow (temperature-induced) photoionization or charge transfer. These non-radiative processes can both greatly diminish *Φ*^31,32^. Therefore, it is essential to ensure that any potential host also has a suitable band gap when screening for new phosphors. These two properties can ultimately be optimized by plotting *Θ*_D_ as a function of DFT calculated *E*_g_, which serves as a sorting diagram. For example, phosphors hosts that fall in the bottom-left corner of this sorting diagram have a low *Θ*_D_ and a narrow *E*_g_, suggesting that they are likely not worth immediate exploration, whereas compounds in the top-right corner of this plot are not only structurally rigid but also have a wide enough band gap to support rare-earth luminescence^20^. The success of this initial sorting diagram has already supported the discovery of numerous inorganic phosphors^5^.

This work establishes a new approach for phosphor screening methods by constructing a more extensive sorting diagram that merges supervised machine learning to predict *Θ*_D_ with high-throughput calculations using DFT for approximating band gap (*E*_g,DFT_). These scalable methods increase the number of potential phosphor hosts contained on the new sorting diagram 50-fold compared to the original approach; therefore, functioning as a more robust guide for phosphor development. Here we use this approach to identify one specific crystal structure that stands out among the 2071 materials on our sorting diagram. NaBaB_9_O_15_ has a high predicted Debye temperature of 729 K, surprising given the mostly ionic bonding and low-density crystal structure, as well as a wide *E*_g,DFT_ of 5.5 eV, making it worthy of experimental investigation. The subsequent synthesis of NaBaB_9_O_15_ substituted with Eu^2+^ indicates that this compound not only has a *Φ* near unity but also shows minimal thermal quenching. These results substantiate the effectiveness of using this sorting diagram to direct the discovery of the next-generation rare-earth substituted inorganic phosphors.

## Results

### Machine learning for predicting Debye temperature and screening inorganic phosphor hosts

The machine-learning model to predict Debye temperature first requires training using a large, diverse set of data. In this case, the 2610 DFT-based bulk (*B*_DFT_) and shear (*G*_DFT_) moduli extracted from the Materials Project database can be converted into an approximate Debye temperature (*Θ*_D,DFT_) based on the average sound velocity (*ν*_m_) following Eq. 1, where *h* is Planck constant, *k*_B_ is Boltzmann constant, *n* is the number of atoms per formula unit, *N*_A_ is Avogadro constant, *ρ* is the crystal structure’s density, *M* is the molar mass, and *ν*_m_ is the average sound velocity^33^:

1 $$\Theta _{{\mathrm{D,DFT}}} = \frac{h}{{k_{\mathrm{B}}}}\left[ {\frac{{3n}}{{4\pi }}\left( {\frac{{N_{\mathrm{A}}\rho }}{M}} \right)} \right]^{\frac{1}{3}}v_{\mathrm{m}}.$$

In a polycrystalline material, *ν*_m_ can be approximated following Eq. 2:

2 $$v_{{\mathrm{m}},{\mathrm{DFT}}} = \left[ {\frac{1}{3}\left( {\frac{2}{{v_{{\mathrm{T}},{\mathrm{DFT}}}^3}} + \frac{1}{{v_{{\mathrm{L}},{\mathrm{DFT}}}^3}}} \right)} \right]^{ - \frac{1}{3}},$$

where *ν*_L_ and *ν*_T_ are the longitudinal and transverse sound velocity, respectively, which can be obtained from the DFT calculated *B*_DFT_ and *G*_DFT_ using Eq. 3:

3 $$v_{\mathrm{L}} = \left( {\frac{{B_{{\mathrm{DFT}}} + \frac{{4G_{{\mathrm{DFT}}}}}{3}}}{\rho }} \right)^{\frac{1}{2}}\;{\mathrm{and}}\;v_{\mathrm{T}} = \left( {\frac{{G_{{\mathrm{DFT}}}}}{\rho }} \right)^{\frac{1}{2}}.$$

**Fig. 1:**
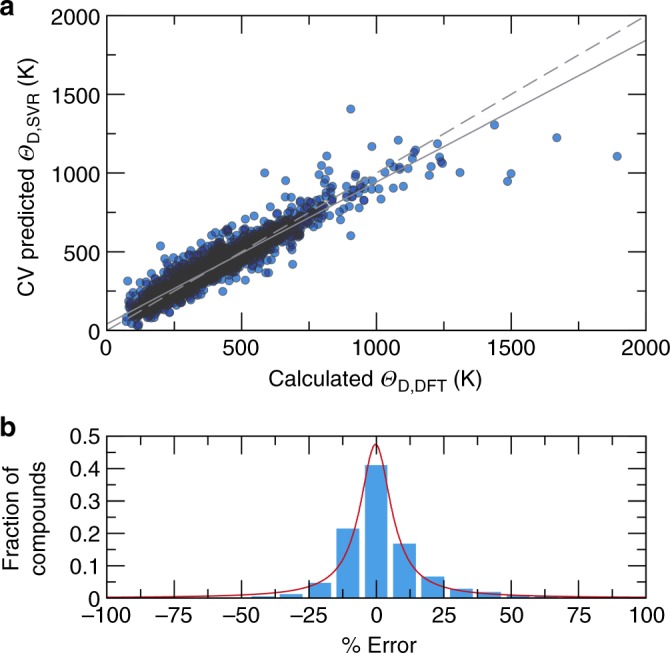
Cross-validation plots for the machine-learning model predicting Debye temperatures. **a** Ten-fold cross-validation (CV) predicted Debye temperature (*Θ*_D,SVR_) versus calculated Debye temperature (*Θ*_D,DFT_). The ideal line is shown as dashed line and the fit line is shown as solid line. **b** Fraction of compounds according to their percent error between CV predicted *Θ*_D,SVR_ and *Θ*_D,DFT_. The red curve shows the trend

Once *Θ*_D,DFT_ was obtained for all 2610 compounds, a machine-learning algorithm was developed to predict the Debye temperature using support vector machine regression (SVR) (*Θ*_D,SVR_). The 10-fold cross-validation regression analysis for *Θ*_D,SVR_ is plotted in Fig. 1a. The statistics of the cross-validated fit show excellent agreement between the *Θ*_D,DFT_ and the *Θ*_D,SVR_ with a root mean-squared error of cross-validation of 59.9 K and a mean absolute error of 37.9 K. The coefficient of determination (*r*^2^) is 0.89 indicating that the descriptor set does an excellent job expressing the materials Debye temperatures. Nearly all of the data (97%) falls between 50 and 750 K, as highlighted by the darker regions of the plot. The cross-validated *Θ*_D,SVR_ tends to be slightly underestimated, particularly for materials with extremely high Debye temperatures (>1500 K), which is potentially due to the lack of data with extremely high Debye temperature. In fact, there are only four compounds, BN, Be_2_C, B_6_O, and LiB_6_C, that fall notably below the expected *Θ*_D,SVR_, suggesting that our machine-learning model does not capture the chemistry of these very light compounds. Although there are a few incorrect predictions, Fig. 1b shows that more than 75% of the compounds are predicted within ≈15% of the DFT calculated value.

**Fig. 2:**
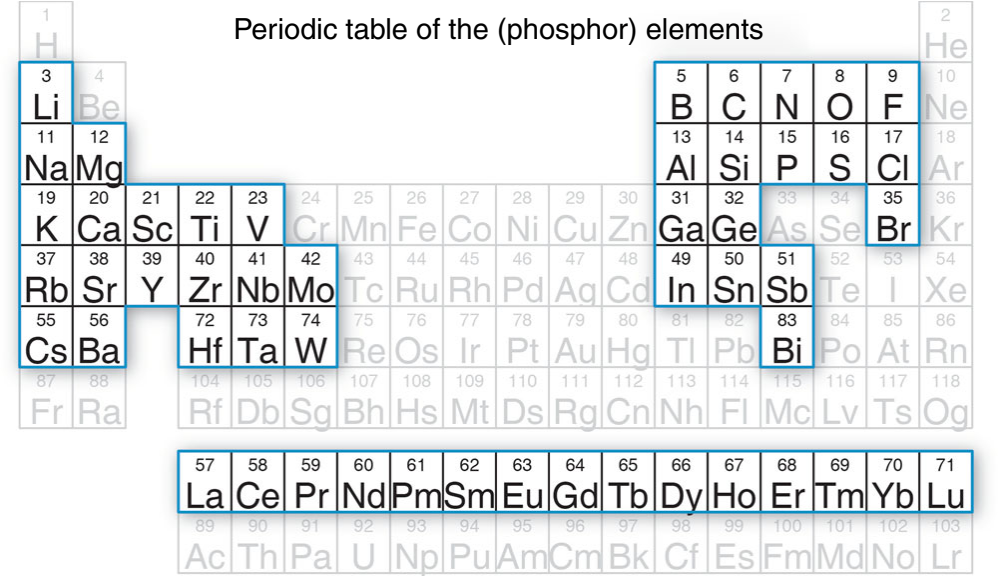
The elements that compose rare-earth substituted phosphor hosts. Potential phosphor hosts were restricted to the highlighted elements. Any compound containing any other element was excluded from the materials screening diagram

Once the training set has been constructed, the machine-learning algorithm can be used to estimate *Θ*_D,SVR_ for compounds compiled in crystallographic databases. The extraction of the crystal structures from PCD followed the same criteria as listed above, supplying 118,287 unique compounds for prediction. This list includes 15,770 binary compounds, 56,266 ternary compounds, and 46,251 quaternary compounds, of which 27,698 (23%) contain rare-earth elements that cannot be readily calculated using conventional DFT and 19,384 (16%) have either disordered positions or site mixing, which is also generally inaccessible with DFT. From this extended list, the search for potential phosphor hosts can be immediately reduced by adhering to a few simple phosphor design criteria. First, knowledge of the band gap is essential for screening phosphor hosts using the sorting diagram, and each compound was further cross-referenced against the Materials Project to ensure that the (Perdew, Burke, and Ernzerhof (PBE)-level) *E*_g,DFT_ is available. Additionally, a majority of rare-earth substituted phosphor hosts contain at least three elements, that is, are ternary phases or higher; therefore, in this study binary compounds are excluded. These two basic requirements reduced the number of possible compounds by 94% to 7504. The list of potential rare-earth substituted inorganic phosphor hosts could be further honed considering rare-earth substituted phosphor hosts are customarily composed of only the elements highlighted by the blue boxes shown in Fig. 2; any compound that does not follow this set of composition requirements was therefore also removed from the list of potential phosphors^10^. Finally, any compound following all of these criteria but still calculated to be a metal, that is, *E*_g_ = 0, was also removed from the list narrowing the final number of possible phosphor hosts to 2071. The *Θ*_D,SVR_ of these 2071 compositions was subsequently predicted with the machine-learning model and associated with the DFT calculated band gap (*E*_g,DFT_). This entire dataset was used to screen potential inorganic phosphor hosts and is provided as Supporting Information.

**Fig. 3:**
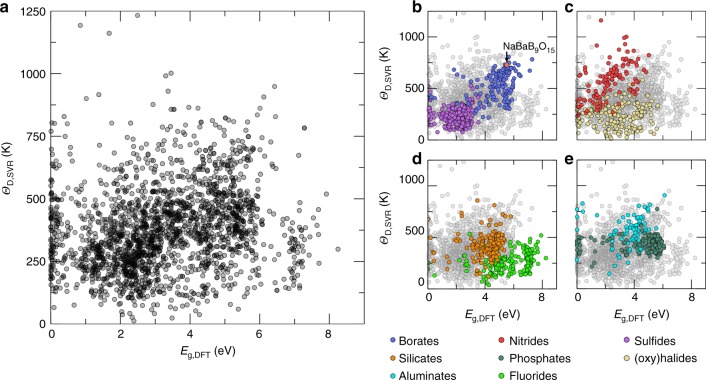
Machine learning predicted Debye temperature against calculated bandgap. **a** Machine learning predicted Debye temperature (*Θ*_D,SVR_) against the density functional theory calculated bandgap (*E*_g,DFT_) for 2071 compounds predicted. The darker regions occur where there are overlapping data. Classes of common phosphor hosts are highlighted, including **b** borates and sulfides, **c** nitrides and (oxy)halides, **d** silicates and fluorides, and **e** aluminates and phosphates

Plotting the 2071 compounds’ *E*_g,DFT_ as a function of their predicted *Θ*_D,SVR_, illustrated in Fig. 3a, creates an expanded sorting diagram that is convenient for balancing these two properties. The darker regions on this plot represent a higher density of potential phosphor hosts. Some (8%) of the compounds on the sorting diagram have very narrow (0 eV < *E*_g,DFT_ ≤ 0.4 eV) PBE-level band gaps, suggesting that these phases are not likely viable options as rare-earth substituted phosphor hosts. There are also a considerable number (22%) with a *Θ*_D,SVR_ <250 K, indicating that these materials may not have the structural rigidity necessary for a high *Φ*.

Decomposing the sorting diagram into the compositional information indicates several trends related to the potential of different phosphor hosts. For example, Fig. 3b shows that borates tend to have wide band gaps and surprisingly high Debye temperatures owing to boron’s small size that allows dense polyhedral packing. Sulfides have a *Θ*_D,SVR_ < 500 K and an *E*_g,DFT_ only in the range of 0–4 eV, which suggests that any emission will likely have a rather low *Φ* when substituted with a rare-earth ion. Conversely, nitrides (Fig. 3c) have moderate band gaps (<4.5 eV), but a generally high *Θ*_D,SVR_ due to the covalently bonded, corner-sharing [SiN_4_] tetrahedra. This allows the predicted Debye temperature to surpass 600 K for many of the compounds and supports the numerous efficient nitride based rare-earth inorganic phosphors. Silicates (Fig. 3d) and aluminates (Fig. 3e), which are perhaps the most common classes of phosphor hosts, tend to have wide band gaps, but they also have a range of prospective *Θ*_D,SVR_ values. Experimentally there are many silicates and aluminates with a high luminescence quantum efficiency, most notably Y_3_Al_5_O_12_:Ce^3+^ or (Ba/Sr)_2_SiO_4_:Eu^2+^, as well as numerous reports of materials that have low luminescence quantum efficiency reinforcing the range of Debye temperatures and band gaps^14,17^. In contrast, fluorides (Fig. 3d) all show very wide band gaps ranging between 4 and 8 eV but low Debye temperatures (<500 K) due to the ionic bonding in these phases. Finally, phosphates (Fig. 3e) tend to have wide bandgaps >4 eV) and moderate Debye temperatures (≈500 K) signifying there is potential for continued development in this space. In fact, a significant number of rare-earth substituted phosphates have been recently reported such as Na_3_Sc_2_(PO_4_)_3_:Eu^2+ ^^15^, BaRbPO_4_:Eu^2+^^34^, and LiSrPO_4_:Eu^2+^^35^, supporting the sorting diagram’s suggestion.

Analyzing metadata-like composition is clearly indispensable for directing the search for new materials, with the potential to shift the search toward remarkable composition space, such as focusing on borates. Indeed, a few high-efficiency borate phosphors have already been reported. For example, Ce^3+^-substituted Ba_2_Y_5_B_5_O_17_ has a blue emission with an *Φ* of 70% at room temperature that can be increased to nearly 90% by substituting the harder cation Lu^3+^ for the comparatively softer Y^3+^^36,37^. Similarly, NaSrBO_3_:Ce^3+^ and NaCaBO_3_:Ce^3+^ both have a reported *Φ* of ≈75%^38,39^. Many of the borate phosphors including K_2_Al_2_B_2_O_7_:Eu^2+^ are also thermally robust showing minimal loss of luminescence at high temperature^40^. Although the search for borate phosphors is directed based on the machine-learning algorithm, the discovery of these materials was still achieved through traditional means including isovalent substitution of known materials, rationally searching phase space, or through serendipitous discovery.

The development of an enhanced sorting diagram also makes it possible to target specific compounds as prospective high *Φ* phosphors. For example, examining the sorting diagram shows that CaAlB_3_O_7_ has a very high *Θ*_D,SVR_ of 732 K and *E*_g,DFT_ of 5.3 eV due to the presence of alternating layers of [AlO_6_] octahedral and [BO_4_] tetrahedra connected through edge and corner sharing, with the Ca^2+^ decorated among the very dense anionic network^41^. Li_5_Rb_2_B_7_O_14_ is also a phase of interest given that *Θ*_D,SVR_ is 703 K and *E*_g,DFT_ is 4.5 eV. This phase is a non-centrosymmetric crystal structure that contains a three-dimensional network of polyborate chains producing the expected rigid crystal structure^42^. Both of these phases are viable phosphor hosts and worth consideration. Additionally, perhaps the most unique crystal structure suggested by the sorting diagram is NaBaB_9_O_15_ (highlighted in Fig. 3b). This compound stands out among the 2071 potential phosphors due to its high *Θ*_D,SVR_ of 729 K and *E*_g,DFT_ of 5.5 eV. NaBaB_9_O_15_ is a particularly remarkable compound because the composition suggests that the bonding should be primarily ionic and not a rigid crystal structure as implied by the *Θ*_D,SVR_. Therefore, experimentally investigating this unanticipated potential phosphor host will lend support to the sorting diagram as a tool for identifying new high *Φ* rare-earth substituted inorganic phosphors.

### Phosphor synthesis and crystal structure refinement

**Fig. 4:**
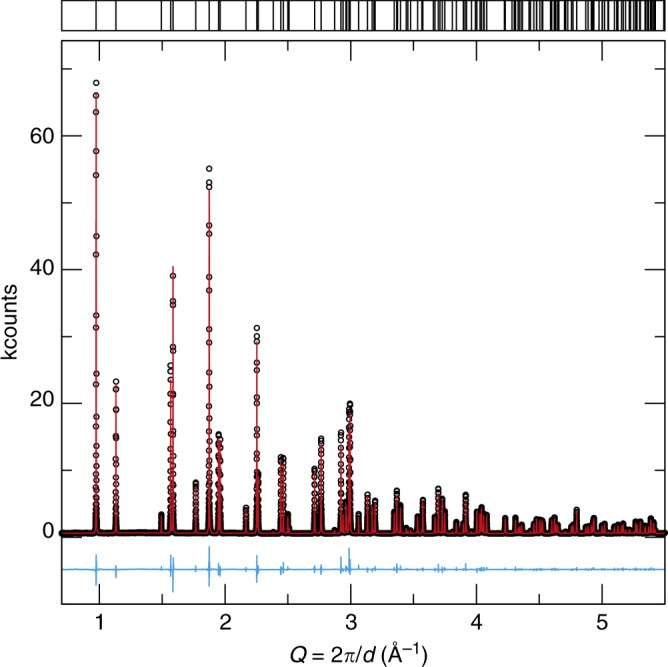
Rietveld refinement of NaBaB_9_O_15_ synchrotron X-ray powder diffraction data. The observed data are colored black, the refinement is colored red, and the difference is colored blue

The synthesis of NaBaB_9_O_15_ yielded a phase pure product according to laboratory (Cu Kα) powder X-ray diffraction. The structure of the unsubstituted, that is, *x* = 0, sample was analyzed using high-intensity, high-resolution synchrotron X-ray powder diffraction data, shown in Fig. 4. The crystal structure was obtained from the Rietveld refinement with details provided in Table 1 and the refined atomic coordinates and isotropic displacement parameters listed in Table 2.

**Table 1:** Rietveld refinement data of NaBaB_9_O_15_

Formula	NaBaB_9_O_15_
Radiation type; *λ* (Å)	Synchrotron radiation; 0.457667
2*θ* range (deg.)	0.5000–49.998
Temperature (K)	295
Space group; *Z*	$R3c$; 6
Lattice parameters (Å)	*a* = 11.10166(6)
	*c* = 17.40089(4)
Unit cell volume (Å^3^)	1857.28(5)
Calculated mass density (g cm^–3^)	2.669
Formula weight (g mol^–1^)	497.595
Profile *R*-factor, *R*_p_	0.0739
Weighted profile *R*-factor, *R*_wp_	0.0907
*χ* ^2^	2.820

**Table 2:** Unit-cell parameters determined using Rietveld refinement

Atom	Wyckoff position	*x*	*y*	*z*	*u*_eq_ (Å^2^)
Ba(1)	6*a*	0	0	0.00101(1)	0.0119(1)
Na(1)	6*a*	0	0	0.22197(9)	0.0152(7)
B(1)	18*b*	0.38819(4)	0.26465(7)	0.05556(1)	0.008(9)
B(2)	18*b*	0.45065(5)	0.39436(4)	0.17207(5)	0.0082(4)
B(3)	18*b*	0.22489(5)	0.32886(5)	0.11364(3)	0.0072(8)
O(1)	18*b*	0.48014(6)	0.32856(7)	0.11349(6)	0.0111(7)
O(2)	18*b*	0.25453(3)	0.24296(1)	0.06014(7)	0.0063(3)
O(3)	18*b*	0.20422(2)	0.4309(3)	0.0678(5)	0.0070(4)
O(4)	18*b*	0.34374(4)	0.41612(6)	0.16374(7)	0.004(1)
O(5)	18*b*	0.1069(8)	0.23238(5)	0.16009(1)	0.0077(8)

**Fig. 5:**
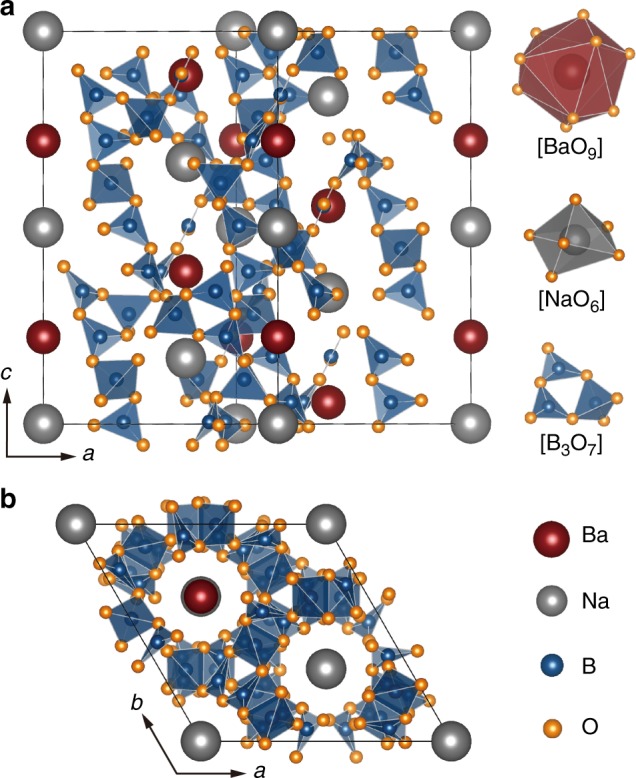
Crystal structure of NaBaB_9_O_15_. **a** Crystal structure of NaBaB_9_O_15_ viewed in [010] direction with the associated [BaO_9_], [NaO_6_], and [B_3_O_7_] polyhedral subunits highlighted. **b** The arrangement of the [B_3_O_7_]^5–^ units generates large tunnels along the [001] direction, which are alternately filled by Ba^2+^ and Na^+^

NaBaB_9_O_15_ adopts the non-centrosymmetric trigonal space group *R3c* (no. 161). As illustrated in Fig. 5, the crystal structure contains a three-dimensional framework of [B_3_O_7_]^5–^ subunits in which two [BO_3_]^3–^ trigonal planar units and one [BO_4_]^5–^ tetrahedron are linked by the vertices. The refined B–O bond lengths within the trigonal planes range between 1.33 and 1.38 Å, while the bond lengths within the [BO_4_]^5–^ tetrahedron are slightly longer (1.45–1.49 Å). The arrangement of the [B_3_O_7_]^5–^ units generates large tunnels along the [001] direction that are occupied by Ba^2+^ and Na^+^. The Ba^2+^ ions are coordinated in a nine-vertex distorted tri-capped trigonal prism, whereas six oxygen atoms form a highly distorted trigonal antiprism surrounding the Na^+^ ions. This combination of corner sharing [B_3_O_7_]^5–^ polyhedra is remarkable because it constructs a network that is not especially dense. In fact, the calculated density of NaBaB_9_O_15_ is only 2.669 g cm^–^^3^ which is half the density of the other reported Na-Ba-B-O phases^43,44^. Such a porous anionic network would not be predicted to form a rigid crystal structure. However, the high *Θ*_D_ likely arises due to the presence of high-frequency vibrational modes from the unique [B_3_O_7_]^5−^ structural unit, which forms an extended and intertwined polyanionic backbone. Moreover, the high concentration of very light boron atoms also gives rise to the presence of high-frequency phonon modes. This combination of composition and crystal structure both contribute to the anomalously high Debye temperature for NaBaB_9_O_15_. Because machine learning has minimal bias, the prediction is undiscriminating in which crystal structures are suggested, highlighting the importance of using this approach to identify phases that may otherwise be disregarded.

Once the crystal structure is refined from high-resolution scattering data, it is possible to experimentally estimate *Θ*_D_ based on the Debye–Waller theory as a confirmation of the machine-learning prediction^45,46^. Although this is a considerable approximation that assumes a single acoustic branch and ignores the optical modes, it is still valuable for comparing the machine-learning predicted Debye temperature to an experimental value. Using the mean-square atomic displacement parameters, <$u_{\mathrm{eq}}^2$>, the *Θ*_D,*i*_ can be calculated for each crystallographically independent atom, *i*, following Eq. 4, where *m* is the atomic mass and *u*_eq,*i*_ is the atomic displacement parameter. The overall Debye temperature (*Θ*_D_) can then be approximated by taking the stoichiometrically weighted average of *Θ*_D,*i*_ according to the formula NaBaB_9_O_15_:

4 $$\Theta _{{\mathrm{D}},i} = \sqrt {\frac{{3\hbar ^2TN_{\mathrm{A}}}}{{m_ik_{\mathrm{B}}u_{{\mathrm{eq}},i}}}}.$$

Evaluating for *Θ*_D_ using the refined <$u_{\mathrm{eq}}^2$> (Table 2) suggests that the Debye temperature should be ≈635 K, which is only 12% lower than the machine learning predicted *Θ*_D,SVR_. This is in an excellent agreement considering that the *B* and *G* values used in the training set are derived from DFT using the Voigt–Reuss–Hill approximation and neglects the temperature dependence of the elastic moduli. Moreover, the scattering refinement data are based on synchrotron X-ray diffraction, which is not particularly adept at evaluating atomic displacement parameters. Nevertheless, the small difference indicates that machine learning is indeed a viable technique for quickly estimating *Θ*_D,SVR_.

### Photoluminescent properties of the identified phosphor

**Fig. 6:**
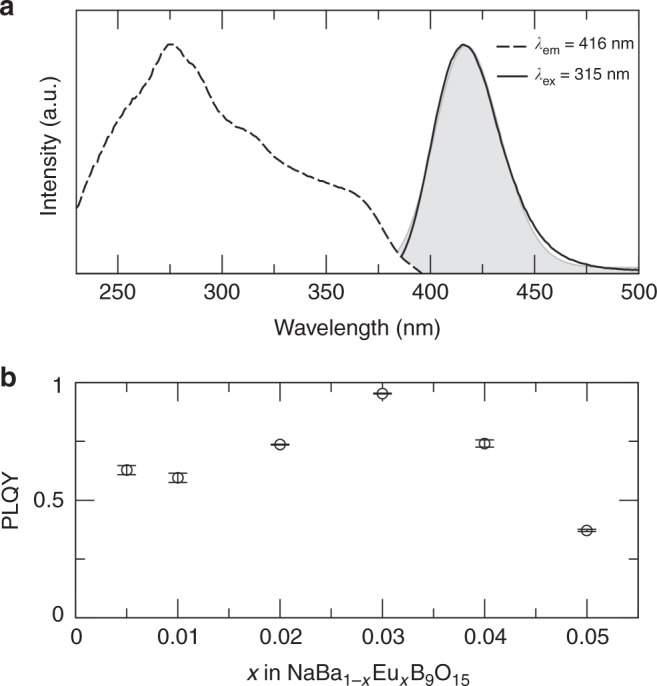
Photoluminescent properties of NaBa_0.97_Eu_0.03_B_9_O_15_. **a** Excitation (dashed line) and emission (black solid line) spectra measured at room temperature. The Gaussian fit of the emission spectrum is solid gray. **b** Measured photoluminescent quantum yield (PLQY) of NaBaB_9_O_15_ substituted with varying concentrations of the rare-earth Eu^2+^ under 315 nm excitation. PLQY for each concentration was measured three times and the error bar represents the standard deviation

The high *Θ*_D,SVR_ and wide PBE-level calculated band gap suggests that NaBaB_9_O_15_ has strong potential as an inorganic phosphor host. Considering that the PBE-level calculations significantly underestimate *E*_g_, a HSE06 hybrid functional calculation was conducted using the Vienna ab initio Simulation Package^47–49^ to ensure that this compound has a sufficient band gap to tolerate the rare-earth’s electronic transitions. The result confirms NaBaB_9_O_15_ indeed has a very wide calculated *E*_g,HSE_ of 7.3 eV. The phosphor was then prepared by substituting Eu^2+^ as the luminescent center for Ba^2+^ following NaBa_1–*x*_Eu_*x*_B_9_O_15_ (*x* = 0, 0.005, 0.01, 0.02, 0.03, 0.04, and 0.05). The compound maintains phase purity across the entire substitution range examined, as plotted in Supplementary Figure 1. Measuring the photoluminescence excitation spectrum indicates that Eu^2+^ has a broad ultra-violet excitation band ranging from 230 to 385 nm, as shown in Fig. 6a. The spectrum contains a main excitation peak centered at *λ*_ex_ = 275 along with two shoulders at *λ*_ex_ = 315 and *λ*_ex_ = 365 nm. The emission spectra collected by exciting at these three wavelengths produces an identical emission peak with a maximum emission wavelength at 416 nm, indicating that the multiple excitation peaks arise from the electronic transitions between the Eu^2+^ 4*f* orbitals to multiple Eu^2+^ 5*d* orbital excited states. The emission spectra can be fit by a single Gaussian peak, depicted in Fig. 6a (and Supplementary Figure 2b), corresponding to the emission from a single crystallographically independent Eu^2+^. This is in agreement with the refined crystal structure that only contains one substitution site, Ba(1). The single Eu^2+^ site was further supported by measuring the photoluminescence lifetime (Supplementary Figure 3). The data could be fit with a single exponential function described in Eq. 5:

5 $$I = I_0 + Ae^{\frac{{ - t}}{\tau }},$$

where *I* is the luminescent intensity, *t* is time, *A* is parameter, and *τ* is the decay time, to reveal that the luminescence decay occurs with a 0.842 μs decay time. The emission is a bright violet with Commission Internationale de l'Eclairage (CIE) 1931 coordinate of (0.1620, 0.01522) and has a very narrow full-width at half-maximum (FWHM) of only 1962 cm^–1^ (34.5 nm). These data show that this material eclipses the industry standard blue-emitting phosphor BaMgAl_10_O_17_:Eu^2+^, which has a FWHM of ≈2200 cm^−1^ (≈50 nm)^50^, but NaBaB_9_O_15_:Eu^2+^ is still broader than the recently reported blue-emitting *AE*Li_2_[Be_4_N_6_]:Eu^2+^ (*AE* = Sr, Ba) phosphors that have a FWHM of only ≈1200 cm^−1^ (≈25 nm)^51^.

Given that the crystal structure of NaBaB_9_O_15_ host is likely a highly rigid phosphor host based on the predicted *Θ*_D,SVR_, the *Φ* of NaBaB_9_O_15_:Eu^2+^ system was extensively analyzed. First, the rare-earth concentration was optimized by varying the loading of Eu^2+^. As shown in Fig. 6b, the 3 mol% Eu^2+^ concentration in NaBaB_9_O_15_:Eu^2+^ has the highest *Φ* = 95(1)%, and increasing the Eu^2+^ concentration causes an abrupt drop in *Φ*. Exciting the phosphor at 340 nm shows the same optimized concentration of Eu^2+^ with a slight decrease in *Φ* to ≈75% with an increase in the rare-earth content beyond 3 mol% again causing a drop in *Φ*. The origin of the emission loss stems from an energy transfer process between adjacent luminescence centers. The critical distance between luminescent centers for this concentration quenching process (*R*_C_) can be calculated following Eq. 6, where *V* is the volume of the unit cell, *x*_c_ is the optimized rare-earth concentration, and *N* is the number of crystallographically independent Eu^2+^ positions in a unit cell^52^. In NaBaB_9_O_15_:Eu^2+^, using a volume of 1857.28 Å, an optimized concentration of *x* = 0.03, and six Eu^2+^ crystallographic sites, the critical distance is approximately 27 Å. Such a large *R*_C_ means that self-quenching is not a very prominent mechanism for non-radiative relaxation in this crystal structure, partly explaining the excellent *Φ*:

6 $$R_{\mathrm{C}} \approx 2\left( {\frac{{3V}}{{4\pi x_{\mathrm{c}}N}}} \right)^{1/3}.$$

**Fig. 7:**
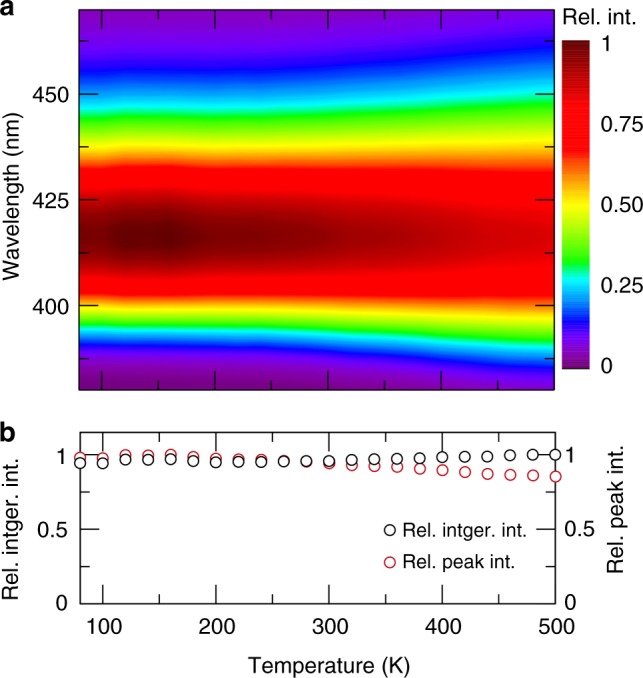
Temperature-dependent photoluminescent properties of NaBa_0.97_Eu_0.03_B_9_O_15_ phosphor. **a** Contour plot of the normalized emission spectra excited at 340 nm as a function of temperature. **b** Relative integrated intensity of the emission spectra (rel. intger. int.) and the relative intensity of the emission peak (rel. peak int.)—as a function of temperature

Beyond concentration quenching, inorganic phosphors can also undergo thermal quenching, where the luminescence intensity is lost due to an increase in non-radiative relaxation. Probing the temperature dependence of the emission spectrum from 80 to 500 K (Supplementary Figure 4) reveals that NaBa_0.97_Eu_0.03_EuB_9_O_15_ possesses an outstanding thermal response. As shown in Fig. 7a, the emission spectrum broadens slightly with increasing temperature going from FWHM = 32.5 nm (1849.5 cm^–1^) at 80 K to FWHM = 40.1 nm (2286.1 cm^–1^) at 500 K. This change can be attributed to increased electron–phonon line broadening at elevated temperatures associated with the excited state 5*d* orbitals of Eu^2+^^5^. More importantly, the emission spectrum at high temperature does not show any thermal quenching, which is the loss of luminescence with increasing temperature, even as the temperature approaches 500 K (Fig. 7b). There is a slight loss of emission intensity once the temperature is >360 K; however, this decrease is only by ≈15%. The integrated area of the emission peak shows no change across the entire temperature range examined because the loss of emission intensity is accompanied by a widening of the FWHM of the emission spectrum, countering the loss of intensity and leading to minimal thermal quenching up to 500 K.

Temperature-dependent powder X-ray diffraction data (Supplementary Figure 5a) indicate that there is no structural modification at elevated temperatures based on refining the crystal structure’s unit cell volume as a function of temperature (Supplementary Figure 5b). Thus, the origin of the outstanding thermal properties in this phosphor can be explained by evaluating the two classical non-radiative pathways that dominate thermal quenching^53^. One process is through thermally induced photoionization, where increasing the temperature can lead to the excited-state electrons occupying the Eu^2+^ 5*d* orbitals being thermally promoted into the phosphor host’s conduction band preventing re-emission. The best method to restrict photoionization is by ensuring that the phosphor host has strong crystal field splitting and a very wide *E*_g_. Separating the rare-earth orbitals from the host conduction band mitigates the probability of thermal excitation at any reasonable temperature, and, therefore, prevents this quenching pathway. Although the crystal field splitting is not considerable, bearing in mind that the borate polyanion is a weak-field ligand, the *E*_g,HSE_ for NaBaB_9_O_15_:Eu^2+^ is still 7.3 eV. This sizeable band gap is likely sufficient to separate rare-earth orbitals from the host conduction band, thereby minimizing this quenching pathway.

The second non-radiative relaxation process occurs via electron–phonon coupling of the ground state and excited state potential energy surfaces^53^. In rare-earth substituted phosphors, the excitation of an electron from the ground state to the excited state causes a simultaneous relative displacement (Δ*r*) of the excited state nuclear coordinates with respect to the ground state nuclear coordinates. If the Δ*r* separating the potential energy surfaces is large, then the two potential energy surfaces can cross (interact), which supplies a mechanism for non-radiative relaxation via electron–phonon coupling^13^. Conversely, in systems where Δ*r* is small, the potential energy surfaces do not interact and the relaxation process can proceed uninhibited, ideally by luminescence. Empirically the best approach for preventing a large Δ*r*, as it relates to changes in bond lengths, might be to target rigid crystal structures^13,54^. However, recent research has showed that *Θ*_D_, as a proxy for rigidity, is not always predictive of thermal quenching^55,56^.

Alternatively, a method to probe Δ*r* in a solid state material is to estimate the electron–phonon coupling by ascertaining a material’s Huang–Rhys factor *S*^57,58^. Experimentally, *S* can be extracted by measuring the FWHM of the rare-earth substituted phosphor’s emission peak as a function of temperature and fitting these data with Eq. 7:

7 $${\mathrm{FWHM}}(T) = \sqrt {8\;{\mathrm{ln}}2} \;\hbar {\mathrm{\omega }}\;\sqrt {S\;{\mathrm{coth}}\frac{{\hbar \omega }}{{2k_{\mathrm{B}}T}}},$$

where ℏω is the mean phonon energy and *k*_B_ is the Boltzmann constant^59,60^. A system that has strong electron–phonon coupling (large Δ*r*) tends to have *S* *>* 5, whereas weak electron–phonon coupling (small Δ*r*) occurs when *S* *<* 1. Fitting the temperature-dependent luminescence data for NaBaB_9_O_15_:Eu^2+^ (Supplementary Figure 6) reveals the Huang–Rhys factor is only 2.098, indicating that this material is approaching the weak electron–phonon coupling regime, and, therefore, should have a relatively small Δ*r*. The consequence is that the two potential energy surfaces in this phosphor are not expected to intersect and the electron–phonon coupling is predicted to be minimal. Thus, this mechanism is also not anticipated to be a primary contributor to non-radiative relaxation in this phosphor. The combination of limited concentration quenching, the absence of photoionization as a quenching mechanism, and the lack of overlap between the ground and excited state potential energy surfaces leads to the robust thermal behavior for NaBaB_9_O_15_:Eu^2+^.

### Conclusions

In conclusion, the Debye temperature of 2071 potential phosphor hosts was predicted with the assistance of machine learning and correlated with their (PBE-level) DFT calculated band gaps. The construction of a sorting diagram based on these two intrinsic material properties is suitable to navigate vast phase space to identify classes of the next generation of inorganic phosphors. For example, borates are worth significant investigation due to their outstanding Debye temperatures and wide (PBE-level) band gaps. One specific borate highlighted by the sorting diagram is NaBaB_9_O_15_:Eu^2+^, which has a *Θ*_D,SVR_ of 729 K and a *E*_g,DFT_ of 5.5 eV. The subsequent synthesis of this phosphor via solid-state reaction showed that the material could be readily prepared as a phase pure product with multiple heating steps. The crystal structure was analyzed using high-resolution synchrotron X-ray powder diffraction and showed a unique polyanionic [B_3_O_7_]^5–^ network, which generates a three-dimensional backbone necessary for a structural rigidity. Substituting the compound with Eu^2+^ yields a violet emission at *λ*_max_ = 416 nm when excited with near-ultraviolet (UV) light (315 nm) light, while the optimal Eu^2+^ concentration produces a maximum *Φ* of 95%. Moreover, the FWHM of the emission is only 34.5 nm, making NaBaB_9_O_15_:Eu^2+^ a narrow violet-emitting phosphor. Temperature-dependent luminescent measurements indicate that this compound is also extremely thermally robust with minimal thermal quenching of up to 500 K due to the lack of non-radiative relaxation pathways. These experimental results support that machine learning is an indispensable tool necessary to direct the search for next generation of rare-earth substituted inorganic phosphors. Although the phosphor discovered in this work is not immediately applicable in current LED-based white lighting devices, it has great potential for use in other applications such as UV-excited laser lighting. Furthermore, the excellent thermal properties of this crystal structure suggest that it is an excellent platform to modify the excitation/emission peaks through chemical substitution. Shifting the optical properties to longer wavelength will make this material viable as a next-generation rare-earth substituted inorganic phosphor.

## Methods

### Data extraction and machine-learning model

To develop the supervised machine-learning model for predicting *Θ*_D_, the bulk modulus *B* and shear modulus *G* of 3248 compounds were obtained from the Material Project database as well as from our own calculations^61^. These values were then cross-referenced with PCD^30^ to ensure that all the compositions used for machine learning are experimentally reported, that is, not a predicted crystal structure. Additionally, only binary, ternary, and quaternary compositions were considered, and any compound containing group 18 elements, hydrogen, Tc, or *Z* > 83 (except for U and Th), were omitted. Finally, thin films, foils, or suspensions were excluded. These criteria reduced the number of elastic moduli in our final training dataset to 2610 compounds, including 1343 binary phases, 1229 ternary phases, and 38 quaternary phases. To distinguish between the polymorphs, the materials were labeled as “composition, space group number.”

The machine-learning model was then constructed based on an SVR analysis using PLS_Toolbox (version 8.2.1) within the MATLAB^®^ (R2017a) environment^62^. The training set employed DFT calculated Debye temperatures obtained as described below. Our SVR applied a radial basis function as the kernel function and was optimized using venetian blinds cross-validation (CV) with 10-fold data splits. The compounds considered by the machine-learning algorithm were evaluated using a descriptor set of 34 distinct compositional variables along with four math expressions including the difference, average, and the largest and smallest values, as well as 14 crystal structure variables such as space group number, crystal system, and electron density, among others. The full descriptor set is provided in Supplementary Table 1. These 150 descriptors (as *x*-vector data) were used to build the machine-learning training model, which was represented in a 2610 × 150 matrix that was normalized, mean-centered, and rescaled to the unit variance. Calculated Debye temperature, *Θ*_D_, was used as *y*-vector data (or dependent variable). This model used an optimized cost parameter (*C*) and gamma (*γ*) functions of 32 and 0.0032, respectively, where *C* regulates the tradeoff between minimization of error and maximization of the margins and *γ* is the kernel parameter controlling the influence of each support vector. This SVR model was then used to predict the Debye temperature of compounds contained within PCD.

### Phosphor synthesis

One phosphor host of particular interest identified in PCD by machine learning is NaBaB_9_O_15_^63,64^. Therefore, NaBa_1–*x*_Eu_*x*_B_9_O_15_ (*x* = 0, 0.005, 0.01, 0.02, 0.03, 0.04, 0.05) was prepared via solid-state reactions starting from NaHCO_3_ (EM science, 99.7%), BaCO_3_ (Johnson Matthey, 98%), H_3_BO_3_ (Sigma-Aldrich, 99.999%), and Eu_2_O_3_ (Materion Advanced Chemicals, 99.9%). The starting materials were loaded in the requisite stoichiometric ratios, thoroughly ground using an agate mortar and pestle, and subsequently sintered at 600 °C for 2 h in air to decompose the reagents and initiate the reaction. The samples were then ground and heated at 700 °C for 15 h, followed by a second grinding and heating at 750 °C for 5 h to obtain a phase pure product. These two heating steps were performed in alumina crucibles using a fused silica tube furnace under a reducing atmosphere (5% H_2_/95% N_2_) with heating and cooling ramps of 3 °C min^–1^. The products were finally ground in an agate mortar and pestle then sieved (<325 mesh) prior to characterization.

### Crystal structure determination and optical characterization

The samples were all checked for phase purity using powder X-ray diffraction on a PanAnalytical X’Pert powder diffractometer equipped with Cu Kα radiation (*λ* = 1.54183 Å). Additionally, powder synchrotron X-ray diffraction data was collected on NaBaB_9_O_15_ at 295 K with a calibrated wavelength of 0.457667 Å (beamline 11-BM, Advanced Photon Source, Argonne National Laboratory)^65^. The crystal structure was refined based on the Rietveld method using the GSAS package with a shifted Chebyshev function employed to describe the background and a pseudo-Voigt function for determining peak shape^66,67^. The final crystal structure was visualized using VESTA^68^.

Steady-state photoluminescent spectra were collected at room temperature on a Photon Technology International fluorescence spectrophotometer with a 75 W xenon arc lamp for excitation. The samples were mixed into an optically transparent silicone resin (GE Silicones, RTV615) and deposited on a quartz substrate (Chemglass) for the room temperature measurements. Temperature-dependent measurements required the sample to be mixed with KBr in a 10:1 molar ratio of KBr:NaBa_0.97_Eu_0.03_B_9_O_15_ and then pressed into a 13 mm pellet. The temperature was controlled using a Janis liquid nitrogen cryostat (VPF-100) and the emission spectra were collected from 80 to 500 K in 20 K increments using a *λ*_ex_ = 340 nm. The absolute (internal) photoluminescent quantum yield was determined by placing the samples encapsulated in the silicone resin inside a Spectralon-coated integrating sphere (150 mm diameter, Labsphere) and exciting at 315 nm^69^. The luminescence lifetime decay measurements were collected using a Horiba DeltaFlex Lifetime System equipped with a NanoLED N-330 nm LED (*λ*_ex_ = 336 nm) and a 400 nm longpass filter. A total measurement length of 3.2 μs was employed using a repetition rate of 250 kHz and a delay of 10 ns.

## Electronic supplementary material

Supplementary Information

Description of Additional Supplementary Files

Supplementary Data 1

## Data Availability

The data that support the findings of this study are available from the corresponding author upon reasonable request.
