# Supplementary material for: Identifying an efficient, thermally robust inorganic phosphor host via machine learning
Source: Nat Commun. 2018 Oct 22;9:4377. doi: 10.1038/s41467-018-06625-z (PMC6197245; doi:10.1038/s41467-018-06625-z)
Supplement: Supplementary file 1 — Supplementary Information [file 41467_2018_6625_MOESM1_ESM.pdf]

Identifying an Efficient, Thermally Robust Inorganic Phosphor Host via Machine Learning

Zhuo et al.

Supplementary Table 1: Descriptor set for predicting the Debye temperature ($\Theta_{D,SVR}$).

descriptor number	compositional variables
1–4	Atomic number
5–8	Atomic weight
9–12	Period number
13–16	Group number
17–20	Family number
21–24	Mendeleev number
25–28	Atomic radius
29–32	Covalent radius
33-36	Zunger radius
37–40	Ionic radius
41-44	Crystal radius
45-48	Pauling EN
49-52	Martynov-Batsanov EN
53-56	Gordy EN
57-60	Mulliken EN
61-64	Allred-Rochow EN
65-68	Metallic valence
69-72	Number of valence electrons
73-76	Gilman number of valence electrons
77-80	Number of s electrons
81-84	Number of p electrons
85-88	Number of d electrons
89-92	Number of outer shell electrons
93-96	First ionization energy
97-100	Polarizability
101-104	Melting point
105-108	Boiling point
109-112	Density
113-116	Specific heat
117-120	Heat of fusion
121-124	Heat of vaporization
125-128	Thermal conductivity
129-132	Heat atomization
133-136	Cohesive energy
137	Space group number
138	Crystal system
139	Laue class
140	Crystal class
141	Inversion center
142	Polar axis

143	Reduced volume
144	Density
145	Average anisotropy (average between a/b, b/c, c/a)
146	Electron density: number of valence electrons per V per Z
147	V per atom
148	Electron density (number of valence electrons per V per atom)
149	Electron density (Gilman valence per V per atom)
150	Electron density (outer shell per V per atom)

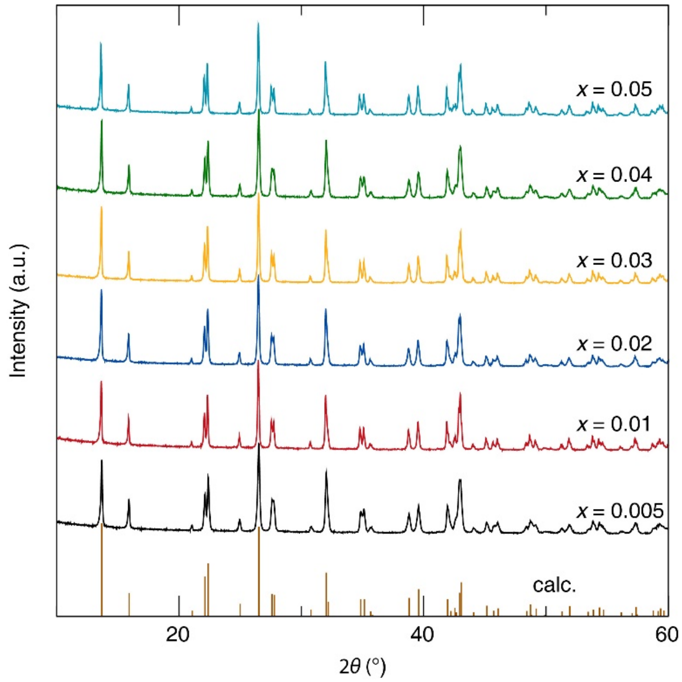

Supplementary Figure 1: Powder X-ray diffraction (XRD) patterns of the $NaBa_{1-x}Eu_xB_9O_{15}$ phosphor. XRD patterns of the synthesized $NaBa_{1-x}Eu_xB_9O_{15}$ (x = 0.005, 0.01, 0.02, 0.03, 0.04, 0.05). All diffraction peaks match well with the calculated pattern of $NaBaB_9O_{15}$ crystal structure.

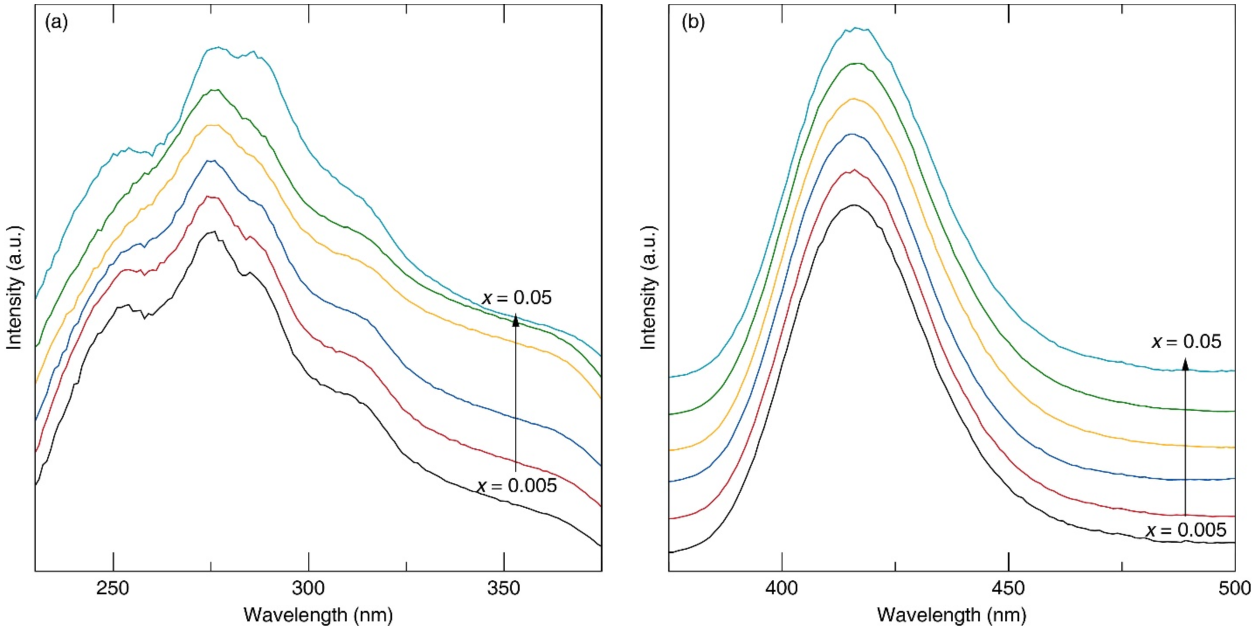

Supplementary Figure 2: Excitation and emission spectra of $NaBa_{1-x}Eu_xB_9O_{15}$ phosphor. Normalized (a) excitation (λ_{em} = 416 nm) and (b) emission (λ_{ex} = 315 nm) spectra for $NaBa_{1-x}Eu_xB_9O_{15}$ (x = 0.005, 0.01, 0.02, 0.03, 0.04, 0.05) at room temperature.

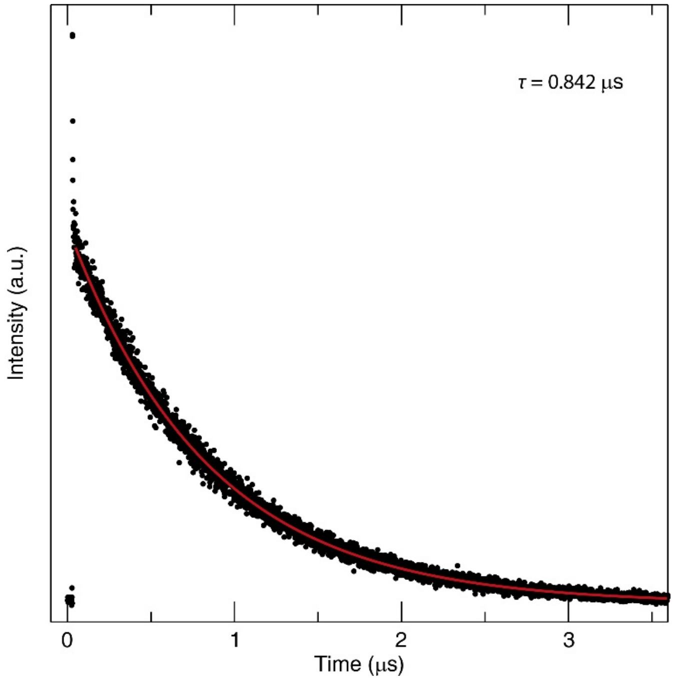

Supplementary Figure 3: Luminescence decay curve of $NaBa_{0.97}Eu_{0.03}B_9O_{15}$. The fit (red line) is obtained following Equation 5 in the main text of the manuscript to obtain the decay time (τ).

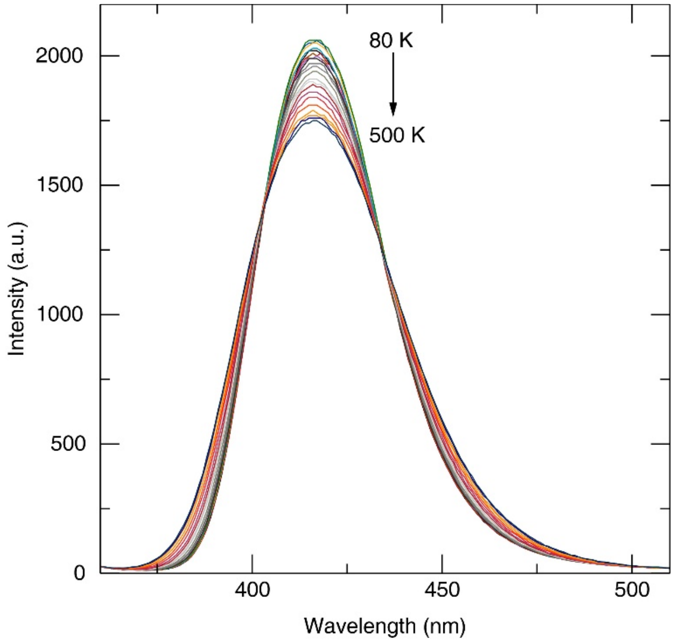

Supplementary Figure 4: Temperature-dependent emission spectra of $NaBa_{0.97}Eu_{0.03}B_9O_{15}$. The data are collected under 340 nm excitation in the temperature range 80–500 K with a temperature interval of 20 K.

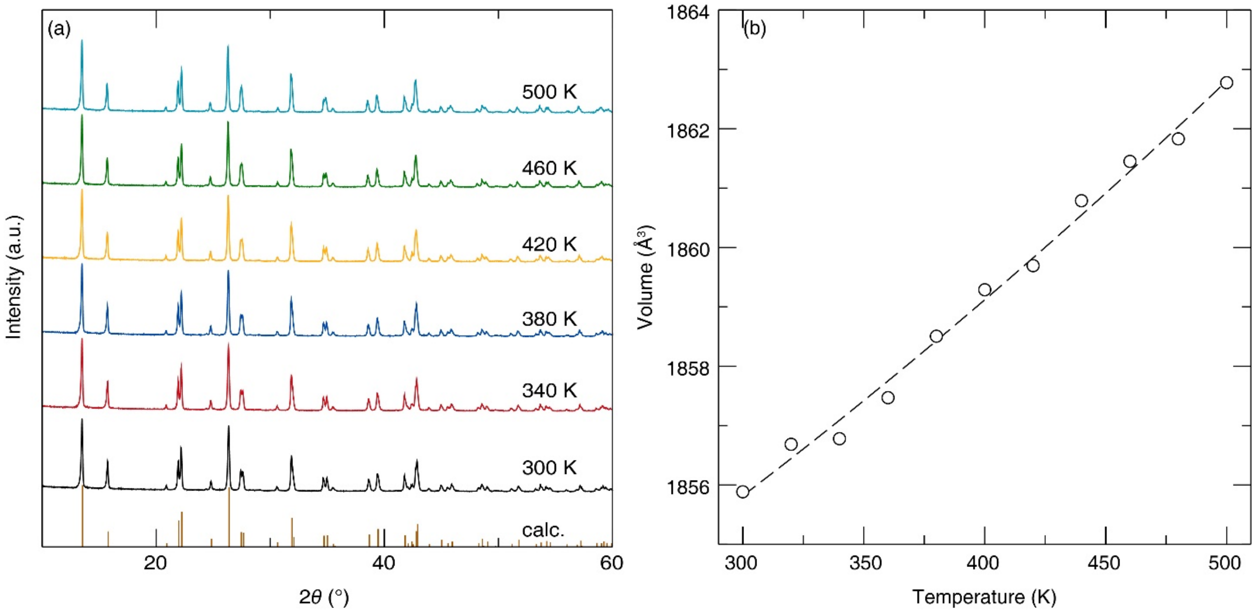

Supplementary Figure 5: Temperature-dependent powder X-ray diffraction patterns of $NaBa_{0.97}Eu_{0.03}B_9O_{15}$ and refined unit cell volume as a function of temperature. (a) The patterns were measured in the temperature range 300–500 K. All diffraction peaks match well with the calculated pattern of $NaBaB_9O_{15}$ crystal. (b) Refined unit cell volume (black circles) obtained with Le Bail method as a function of temperature, which is fit by a second order function (dashed line).

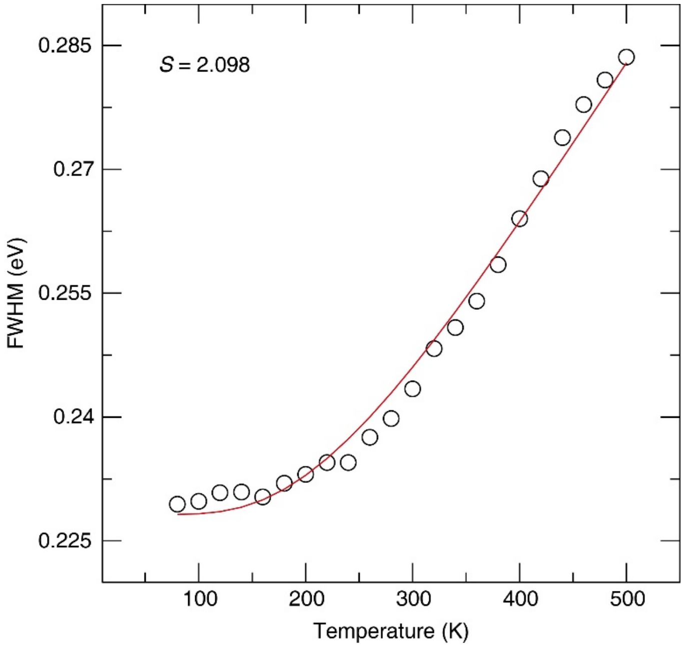

Supplementary Figure 6: Temperature dependence of the full width at half maximum (FWHM) of the emission peak. The data points (black circles) are extracted by fitting the experimental emission data and the fit (red line) is obtained following Equation 7 in the main text of the manuscript to obtain the Huang-Rhys factor, *S*.
