# Supplementary material for: Identifying an efficient, thermally robust inorganic phosphor host via machine learning
Source: Nat Commun. 2018 Oct 22;9:4377. doi: 10.1038/s41467-018-06625-z (PMC6197245; doi:10.1038/s41467-018-06625-z)
Supplement: Supplementary file 2 — Description of Additional Supplementary Files [file 41467_2018_6625_MOESM2_ESM.pdf]

Description of Additional Supplementary Files

File Name: Supplementary Data 1

Description: The chemical composition (Column A), associated space group number (Column B), the density functional theory calculated bandgap (PBE-level) (Column C), and the support vector regression predicted Debye temperature for 2071 compounds compiled in the Pearson's Crystal Database (Column D). These data were employed for constructing the sorting diagram presented in the main text.
